# Diseases Caused by and Behaviors Associated with *Toxoplasma gondii* Infection

**DOI:** 10.3390/pathogens13110968

**Published:** 2024-11-06

**Authors:** Ginger K. H. Akins, João M. Furtado, Justine R. Smith

**Affiliations:** 1 College of Medicine and Public Health, Flinders University, Bedford Park, Adelaide, SA 5042, Australia; akin0035@flinders.edu.au; 2 Division of Ophthalmology, Ribeirão Preto Medical School, University of São Paulo, Ribeirão Preto, São Paulo 14049-900, Brazil; furtadojm@gmail.com; 3 Flinders Health and Medical Research Institute, Flinders University, Bedford Park, Adelaide, SA 5042, Australia

**Keywords:** *Toxoplasma gondii*, ocular toxoplasmosis, cerebral toxoplasmosis, congenital toxoplasmosis, personality, risky behavior, schizophrenia, bipolar disorder

## Abstract

*Toxoplasma gondii* is an Apicomplexan parasite that is estimated to infect at least one-third of the global human population. *T. gondii* infection may be transmitted horizontally or vertically. The main risk factors for transmission to humans are related to diet, especially the consumption of undercooked meat, along with soil contact. In immunocompetent persons, the acute infection may go undetected as it typically produces minor, non-specific symptoms that are self-limited. After infection is established, recurrent retinochoroiditis is the most common clinical disease. In contrast, severe systemic or cerebral toxoplasmosis may be life-threatening for immunocompromised individuals. Furthermore, congenital toxoplasmosis acquired in utero may have devastating consequences if not recognized and promptly treated. A growing body of research has identified associations between latent *T. gondii* infection, and personality traits and risk-taking behaviors. Other studies have documented associations between latent infection and psychiatric conditions that include schizophrenia and bipolar affective disorder. With no current treatment regimens being curative of *T. gondii* infection, effective prevention measures at both the public health and individual levels are vitally important.

## 1. Introduction

*Toxoplasma gondii* infects approximately 30% of the human population, causing a spectrum of diseases broadly known as toxoplasmosis. Beyond clinical conditions that include cerebral toxoplasmosis, ocular toxoplasmosis, and congenital toxoplasmosis, there has been recent interest in the behavioral and neuropsychiatric associations of the infection. This comprehensive review summarizes the full range of potential consequences of the human infection with *T. gondii*, including diseases, behavioral traits, and mental illnesses. The article begins with a discussion of *T. gondii* parasitology, continues with descriptions of the diseases caused by infection including their clinical features and diagnostic investigations, followed by psychiatric illnesses and behaviors associated with infection, and ends with summaries of treatment approaches and preventive measures.

## 2. Parasitology

### 2.1. Parasite Life Cycle

*Toxoplasma gondii* is a protozoan parasite from the phylum Apicomplexa, with a complex lifecycle that can be divided into sexual and asexual stages [1]. The sexual stage of the *T. gondii* lifecycle only occurs in animals of the Felidae family, including domestic and wild cat species, making these the definitive hosts [1]. During the asexual stage, *T. gondii* has the capacity to infect any warm-blooded intermediate host, including humans [1,2]. Throughout these lifecycles, *T. gondii* has different infective forms [1]. Tachyzoites are rapidly replicating forms present during acute infection and reactivations, while bradyzoites are dormant forms that undergo minimal replication and are characteristic of chronic infection [1,3]. Within the host tissue, bradyzoites convert into cysts that are resistant to eradication by both immune responses and current drug therapies [1,3,4]. Sporozoites are the product of the sexual lifecycle in the definitive host and are dormant, but highly infectious, forms that are released into the environment in oocysts via the feces [1,3,4].

Felid consumption of *T. gondii* in any of its infectious forms initiates the sexual stage of the life cycle, although the consumption of bradyzoite cysts in infected meat from an intermediate host is better described [1]. Once ingested, bradyzoites are released from cysts within the small intestine, where they infect local enterocytes and undergo asexual reproduction [2]. The ultimate products are microgametes and macrogametes, which fuse together during fertilization to produce an immature oocyst [1,2]. The immature oocyst is released back into the felid intestinal tract and excreted via the feces [1]. Sporulation only occurs upon exposure to the external environment and results in a mature oocyst containing eight sporozoites that is able to withstand months of variable external conditions [1].

The asexual stage begins when an intermediate host is infected, typically by ingesting a mature oocyst from contaminated water or food, or a tissue cyst in raw meat [1]. Oocysts and tissue cysts are digested in the small intestine to release sporozoites and bradyzoites, respectively [2]. Both forms of *T. gondii* infect enterocytes lining the intestinal tract, replicating and transforming into tachyzoites, which move from the intestines to disseminate throughout the body via blood and lymphatic vessels [2]. Any nucleated cell can be infected by tachyzoites and, as a result, *T. gondii* can infect a range of tissue types [1]. In humans, it is thought that the infection of leukocytes responding to inflammation caused by the initial infection of enterocytes is particularly important in the dissemination of tachyzoites to distant tissues [5,6]. Bradyzoites tend to persist in the central nervous system (CNS), an immune-privileged site ideal for supporting chronic latent infection [7,8]. However, the route by which tachyzoites crosses the blood–brain barrier (BBB) to infect the CNS is not fully understood [9]. Experiments conducted with human retinal endothelial cells suggest three possibilities: (1) in dendritic cell taxis; (2) as free parasites that navigate through the endothelium; and (3) through infection of the endothelial cells, which have high susceptibility [10,11,12].

At the cellular level, *T. gondii* infection involves three stages that form a lytic cycle: invasion, replication, and egress [13]. Tachyzoite invasion is an active process that relies upon actin–myosin-mediated gliding via a specialized protein complex called a glideosome [13,14]. Proteins are released from the apical end of the tachyzoite, mediating its attachment to the host cell [14]. The glideosome then allows the tachyzoite to progress forward into the host cell, forcing the plasma membrane to invaginate and engulf the parasite within an internalized vesicle called the parasitophorous vacuole [14]. Within the parasitophorous vacuole, the tachyzoite manipulates the host cell to promote its own growth and replication, while inhibiting host cell death, until the replicative phase is completed [15]. Replication occurs inside the parasitophorous vacuole via endodyogeny [14]. This involves the production of two daughter progeny within the parent tachyzoite, which is eventually consumed and replaced by two daughter parasites [13,14]. After variable rounds of asexual replication, the final stage of the lytic cycle, egress, occurs as both the parasitophorous vacuole and host cell membranes are ruptured to release the multiplied daughter tachyzoites [16]. Egress is an active stage and is mediated by glideosome motility, along with the release of various parasite and host cell-derived proteins [16]. Primary among these are parasite perforin-like protein TgPLP1, which creates pores within the parasitophorous vacuole and host cell plasma membranes, and host cell calpain 1, which restructures the cytoskeleton [16]. Egress of the daughter tachyzoites results in the death of the host cell and allows for the continuation of the lytic cycle as new cells become infected to produce more parasites [2]. Within target tissues, tachyzoites continue to undergo asexual replication in this manner until the host immune system triggers a transformation into dormant, encysted bradyzoites [2].

### 2.2. Prevalence of Human Infection

Humans are intermediate hosts for *T. gondii* and, globally, approximately 30% of the human population is infected with this ubiquitous parasite [13,17]. However, there is great variation in infection rates between countries, ranging from 0.5% to 87.7% [17], and even between populations within countries [18]. According to a systematic review from 2019, despite the shortage of epidemiological studies in some areas of the world, African countries had the highest average reported seroprevalence rate of 61.4% (range 20.8–87.7%), while Asian countries reported the lowest average seroprevalence rate of 16.4% (range: 0.8–82.6%) [17]. The average reported seroprevalence of *T. gondii* in Europe was 29.7% (range: 8.2–59%), in the USA/Canada the rate was 17.5% (range: 10.4–22.5%), in South America the rate was 31.2% (range: 7.3–74.7%), and in Oceania the rate was 38.5% (range: 23–60%) [17].

### 2.3. Sources of Human Infection

The substantial variation in the prevalence between regional populations can be attributed in large part to the factors that affect human infection of *T. gondii,* which occurs via vertical or horizontal transmission [19]. Horizontal transmission most commonly occurs following the consumption of either oocysts in contaminated food or water, or bradyzoite cysts in raw or undercooked meat [20]. Vertical transmission occurs after a primary infection in a pregnant mother, when tachyzoites cross the placenta to infect the fetus [21]. Rare routes of horizontal transmission include contaminated blood transfusions or organ transplants [22]. 

#### 2.3.1. Meat

Diet and the consumption of contaminated food is thought to be the most important risk factor for the transmission of *T. gondii* infection [23,24,25]. In particular, the consumption of raw or undercooked meat has been consistently shown to increase the risk of infection, with 47.1% of acute toxoplasmosis outbreaks linked to the consumption of contaminated meat in one systematic review [23,24,25,26,27,28]. However, there is less consensus in the literature on the associations of different types of livestock with transmission [23,24,25]. A systematic review including studies from all major global geographical areas found that the pooled *T. gondii* prevalence was greatest in sheep (14.7%), followed by pigs (12.3%), and then cattle (2.6%) [23]. This systematic review also highlighted the great heterogeneity in prevalence according to the geographical location of the livestock, likely explaining the varied findings in the literature [23]. Small goods, which generally are prepared from multiple carcasses, are more likely to harbor *T. gondii* than whole cuts of meat [29]. 

#### 2.3.2. Water

*T. gondii* oocysts are environmentally resistant, remaining infective for up to 18 months in water [30]. Water contaminated with oocysts is of particular concern to public health due to the potential to rapidly expose large populations [31]. One systematic review analyzed studies from around the world and found that *T. gondii* detection rates ranged from 2.3% to 17.9% in piped water used in homes or for public drinking [30]. Furthermore, another systematic review found that 20.6% of all acute toxoplasmosis outbreaks from 1967 to 2018 were linked to the ingestion of oocysts from contaminated water [28,32]. The largest outbreak of human toxoplasmosis to date was reported in Santa Maria, South Brazil, in 2018, when over 900 individuals were infected with *T. gondii* through contaminated drinking water [33]. It is also postulated that the accidental ingestion of contaminated recreational water, such as from rivers and lakes, may cause some infections [31].

#### 2.3.3. Soil

*T. gondii* cysts can remain infective in soil or sand for up to 2 years [30]. According to the location of the soil, different detection rates have been estimated, ranging from 1.1% in playgrounds up to a concerning 94.1% in urban vegetable gardens [30]. Contact with soil has been identified as a significant risk factor for human infection with *T. gondii* in multiple studies, with up to 17% of *T. gondii* infections in pregnant women across Europe and an estimated 17.6% of acute toxoplasmosis outbreaks attributed to contact with soil or sand [24,28,34,35,36].

#### 2.3.4. Fruit and Vegetables

The surface of fruit and vegetables can become contaminated via water or soil containing cat feces with oocysts. Indeed, one study isolated *T. gondii* DNA from 40% of the fresh fruit and vegetable collected from local suppliers and supermarkets across Spain and Portugal, showing a much higher prevalence than previous studies [37]. There is limited evidence definitively linking contaminated fruits and vegetables to confirmed cases of *T. gondii* infection, although a study in mice demonstrated that transmission was possible by showing that the consumption of berries coated with oocysts led to infection [38]. Furthermore, two outbreaks of acute toxoplasmosis in Brazil in 2009 and in 2013 were linked to contaminated vegetables and Acai, respectively [28,39]. These cases were included in a systematic review, which estimated that 5.9% of acute toxoplasmosis outbreaks from 1967 to 2018 were attributed to the consumption of raw vegetables and fruit.

#### 2.3.5. Contact with Cats

As cats are the definitive host of *T. gondii*, it has long been suggested that increased interaction with cats may increase the risk of human infection. However, the oocysts shed in cat feces are initially unsporulated and take days to mature into the infectious, sporulated form [18]. This means that contacts with cats themselves is less likely to result in human infection, as supported by a study in Naples, which found that recent *T. gondii* infection in pregnant women was not associated with cat ownership [27]. While most studies corroborate that cat ownership itself is not a risk factor for *T. gondii* infection in humans [18,24,25,27], contact with cat feces is more controversial. One case–control study in Europe found that neither contact with cats nor contact with cat feces were risk factors [24], while another case–control in Norway found that cleaning a cat litter box was strongly associated with infection [25]. Despite these conflicting reports, it is generally recommended that vulnerable individuals avoid contact with cat feces [18,25].

## 3. Diseases Caused by Infection

### 3.1. Acute Systemic Infection

Acute systemic infection with *T. gondii* typically occurs during dissemination of tachyzoites from a primary infection. The clinical presentation of this acute systemic infection is mostly dependent upon the competency of the infected individual’s immune system [3,19,40]. 

#### 3.1.1. Immunocompetent Individuals

In immunocompetent patients, the parasite triggers a robust immune response and in approximately 90% of cases, the acute infection is asymptomatic [3,19,40]. In symptomatic patients, the vast majority experience mild, non-specific, and self-limited symptoms [19]. The most common presentation is a non-tender lymphadenopathy in the cervical region, typically reducing within 6 weeks [41]. In rare cases, more serious disease, such as retinitis, encephalitis, hepatitis, myocarditis, or pneumonitis, can occur from the primary infection [19,41]. While immunocompetent patients are usually able to control the acute infection without showing overt symptoms, the immune system is unable to eradicate the parasite, which differentiates from active tachyzoites into dormant bradyzoites, marking the transition to the chronic phase of infection [3,40].

#### 3.1.2. Immunocompromised Individuals

In immunocompromised individuals, such as individuals infected with human immunodeficiency virus (HIV), patients with cancer, persons taking immunosuppressive drugs, and organ donor recipients, primary *T. gondii* infection can result in more serious disease, as rapidly proliferating tachyzoites damage essential organs [3,42]. The clinical presentation of toxoplasmosis in immunocompromised patients is dependent upon the site of tissue damage, with the most common presentations being cerebral toxoplasmosis and ocular toxoplasmosis [19,42].

### 3.2. Cerebral Toxoplasmosis

Cerebral toxoplasmosis refers to active *T. gondii* infection of the brain. Although it is debated whether *T. gondii* displays tropism for the CNS during the acute phase of infection, the parasite is still considered neurotropic as the bradyzoite cysts that form during chronic infection persist in higher loads within the immune-privileged CNS compared to peripheral tissues, from which cysts can be cleared over time [1]. Thus, reactivation of the parasite in immunocompromised patients is more likely to result in infection of the CNS [7]. Risk factors for developing cerebral toxoplasmosis among immunocompromised individuals include a CD4+ T cell count of below 200/μL, poor control of HIV infection, and lack of prophylactic treatment to prevent toxoplasmic encephalitis [42,43,44].

#### 3.2.1. Clinical Manifestations

The symptoms of cerebral toxoplasmosis vary according to the site and number of lesions within the brain [19,42,45,46]. The most common manifestation of cerebral toxoplasmosis is necrotizing encephalitis, resulting in multiple focal lesions within the brain [45,46]. Less commonly, cerebral toxoplasmosis can present as a generalized encephalitis or ventriculitis [19,42]. Studies have shown that the most common symptoms of cerebral toxoplasmosis are headache, fever, change in mental status, seizures, and a range of focal neurological deficits [19,42,45]. While cerebral toxoplasmosis can cause encephalitis, the meninges are usually spared and, as such, the classic signs of meningism are rarely seen [45]. Symptoms of cerebral toxoplasmosis usually present over a subacute time course, although a rapidly progressive and even fatal version of the disease can occur [45].

#### 3.2.2. Serological and Radiological Diagnostic Investigations

As cerebral toxoplasmosis can mimic other brain lesions, investigative tests are often needed to confirm a diagnosis [42]. Serological confirmation of infection can be achieved by detecting the presence of anti-*Toxoplasma* immunoglobulin (Ig)M and IgG. The gold standard test is the Sabin–Feldman dye test, although enzyme-linked immunosorbent assays (ELISAs) are more practical and are therefore more commonly used. Anti-*Toxoplasma* IgM is usually present within one week of primary infection, and can remain detectable for over 12 months, while IgG normally peaks around 2 months following acute infection and persists for the remainder of the patients life [42]. The presence of IgM and no detected IgG likely indicates a recent primary infection, while the presence of IgG with no detected IgM generally excludes recent infection. However, the possible persistence of IgM for over one year makes it much more difficult to comment on the timing of infection if both IgM and IgG are detected. Through modification of the ELISA, antibody avidity can be determined, and as avidity increases over time after the initial infection, these values can help predict when the primary infection occurred [42]. 

Suggestive clinical features and positive serological tests are often not specific enough to diagnose cerebral toxoplasmosis, necessitating the use of neuroimaging. Magnetic resonance imaging (MRI) has the greatest sensitivity for diagnosing cerebral toxoplasmosis and is the preferred modality, but computed tomography (CT) is often a more practical option in practice [45,47]. Characteristically, a CT of the brain shows multiple, ring-enhancing lesions with a mass effect caused by the surrounding edema [42,45,46]. Other CT patterns associated with cerebral toxoplasmosis include a singular ring-enhancing lesion, nodular-enhancing lesions, non-enhancing lesions, and even diffuse cerebral edema with no focal lesions [45]. However, MRI is often able to show lesions in cases where CT has detected only one or no lesions [42]. On contrast-enhanced MRI, the characteristic signs of cerebral toxoplasmosis are eccentric and concentric target lesions [42,45]. These lesions can be found in any region of the brain, although they are most often seen within the basal ganglia, the thalami, and the junction between gray and white matter [42,45,46].

### 3.3. Ocular Toxoplasmosis

Ocular toxoplasmosis is an umbrella term for eye disease as a result of *T. gondii* infection and is acknowledged as the most common clinical presentation of toxoplasmosis [48]. The prevalence of this condition has been estimated at 0.67% to 5.8% in community-based surveys from different parts of the world [49]. Ocular toxoplasmosis is the most common cause of posterior uveitis, or in some populations, of all types of uveitis [50]. Unsurprisingly given its prevalence, ocular toxoplasmosis has a significant disease burden, as demonstrated by a study in the Netherlands which found that the combined disability-adjusted life years (DALYs) for congenital and postnatally acquired toxoplasmosis was 2400 DALYs [51].

#### 3.3.1. Clinical Manifestations

While ocular toxoplasmosis is a common manifestation of congenital infection, the majority of cases are a result of postnatal *T. gondii* infection [52,53]. Patients normally present with ocular toxoplasmosis when relatively young compared to non-toxoplasmic cases of uveitis, with a mean age of approximately 27 years old [54]. The location and size of the typical retinal lesion influences the symptoms experienced, as small or peripheral lesions may be asymptomatic while involvement of the macula can cause significant visual disturbance [48,52,55]. The impact upon vision has been demonstrated in large prospective studies that document around one in five patients with toxoplasmic retinochoroiditis suffers from severe vision impairment (Snellen visual acuity of 20/200 or less) in the affected eye [55,56]. Further to this, retinal lesions as a result of congenital toxoplasmosis are more likely to be located at the macula and are much more likely to be bilateral [48,57]. Therefore, congenital ocular toxoplasmosis has a greater impact upon vision, as shown in a retrospective, cross-sectional study that found all 17 patients with severe congenital toxoplasmosis went on to develop retinal lesions, and of these patients 85% had a visual acuity less than 20/200 [58]. Adults or verbal children may report altered vision, floaters or ocular pain, while in preverbal children, leukocoria or strabismus may be observed in the affected eye [48,59]. Nystagmus has also been reported in patients with both maculae affected and is more common in congenitally acquired ocular toxoplasmosis [48,57].

Clinically, in the vast majority of patients, ocular toxoplasmosis presents as a unilateral, focal necrotizing retinochoroiditis [54]. Active toxoplasmic retinochoroiditis classically appears as a fluffy white lesion that spans all layers of the retina [59,60,61]. According to the size of the lesion, there is variable secondary inflammation of the choroid and vitreous [59,60]. Severe vitritis can produce the classic “head light in the fog” sign [59]. In immunocompetent patients, the active lesion typically resolves within 4 months, leaving a hyperpigmented retinal scar [62]. Unless it is the initial primary lesion, the active lesion is normally located adjacent to a previous retinal scar [60]. While this retinochoroiditis is by far the most common presentation of ocular toxoplasmosis, many atypical presentations have been described in the literature, including but not limited to punctate outer retinal toxoplasmosis, rhegmatogenous and serous retinal detachments, anterior uveitis, and scleritis [59,60,63]. 

#### 3.3.2. Serological and Ocular Diagnostic Investigations

The characteristic appearance of toxoplasmic retinochoroiditis is often considered sufficient to confirm ocular toxoplasmosis [64]. However, several investigations may be useful in the setting of atypical or particularly severe presentations [59,65]. Serological testing (see “*Cerebral toxoplasmosis*”) is a useful initial investigation to rule out *T. gondii* infection. As *Toxoplasma* IgG are generally present for life after initial infection, their absence can be used to exclude the disease in immunocompetent patients, although false negatives can occur in immunocompromised patients [19,65]. However, due to the high seroprevalence in the population, a positive serological result, even when combined with positive clinical findings, cannot always confirm a diagnosis of ocular toxoplasmosis [65]. The direct testing of ocular fluid can assist in diagnostically challenging cases and includes the polymerase chain reaction (PCR) for *T. gondii* DNA or calculating the Goldmann–Witmer Coefficient (GWC) by comparing intraocular anti-*Toxoplasma* antibody titers to serum titers. In general, the GWC has greater sensitivity while PCR is highly specific, approaching almost 100% specificity if contamination is eliminated, although the greatest accuracy has been demonstrated when both tests are combined [19,52,66]. Immunoblot or Western blot may be used as an alternative to the GWC [66].

### 3.4. Congenital Toxoplasmosis

*T. gondii* infection acquired during pregnancy is of particular concern due to the risk of transmission to the fetus, resulting in congenital toxoplasmosis. Almost all congenital toxoplasmosis is a result of maternal infection acquired during pregnancy [67,68,69,70]. However, in rare cases, transmission has been reported in mothers infected prior to conception, with 3 months being considered to be the longest time a primary infection can occur prior to conception and result in congenital toxoplasmosis [67,68,69,70]. Another rare scenario of congenital toxoplasmosis with ocular and cerebral manifestations in consecutive siblings has been reported [71]. The global incidence of congenital toxoplasmosis has been estimated as 1.5 cases per 1000 live births (95% confidence interval = 1.4–1.6), resulting in a burden of 1.2 million DALYs [72]. The overall risk of transmission from an infected mother to the fetus is estimated as 29%, based on a study in France, where there is a compulsory national screening program for recent *T. gondii* infection in pregnant women [73]. However, the timing of maternal infection in relation to the stage of pregnancy affects the rate of transmission, with one study demonstrating that the risk of infection increased from 6% at 13 weeks to 72% at 36 weeks [73,74].

#### Clinical Manifestations

An earlier gestational age at the time of maternal infection is associated with a more severe clinical presentation [73]. Furthermore, systematic reviews indicate that more virulent or atypical *T. gondii* strains, lower socioeconomic status, and delays in anti-parasitic treatment are associated with worse outcomes [74,75]. Age of clinical presentation also aligns with gestational age at maternal infection, with clinically apparent disease present in 77.8% of newborns whose mothers were infected in the first trimester (22.2% subclinical disease) compared to 10.2% of newborns whose mothers were infected in the third trimester (89.8% subclinical disease) [19]. The time intervals between birth and diagnosis, and birth and treatment also influence symptom presentation: a Brazilian study showed that starting antiparasitic drugs within the first 4 months of life reduced the risk of developing new retinochoroidal lesions after the first year of life to 35.2%, compared to 77.8% in patients who began treatment after 4 months [76]. 

Congenital toxoplasmosis presents clinically at different times during the patient’s life and can be divided accordingly: (1) in utero, (2) clinically apparent at birth, (3) subclinical at birth, and (4) late manifestations.

1. Congenital toxoplasmosis in utero

As many as two-thirds of fetuses with congenital toxoplasmosis shows no abnormalities on ultrasound scans [19,77]. If present, sonographic signs include intracranial calcifications, hydrocephalus, ventricular dilation, hepatic enlargement, ascites, and intrauterine growth restriction [19,77]. Furthermore, maternal *T. gondii* infection during pregnancy is significantly associated with an increased risk of spontaneous abortion and is also suggested as a risk factor for stillbirth, as supported by many animal studies [78,79,80,81,82,83].

2. Clinically apparent congenital toxoplasmosis at birth

It is estimated that between 10% and 30% of infants with congenital toxoplasmosis have clinically apparent symptoms at birth, and the majority of these are linked to infection in the first trimester of pregnancy [19,84,85]. Historically, a triad of retinochoroiditis, hydrocephalus, and intracranial calcifications has been touted as the classic sign for congenital toxoplasmosis apparent at birth, although in practice the prevalence of this triad is variable among different populations, ranging from less than 10% to 62% in different studies [86,87,88]. The various other clinical signs of congenital toxoplasmosis apparent at birth include jaundice, thrombocytopenia, anemia, hepatosplenomegaly, seizures, skin rashes, microphthalmia, and microcephaly [89].

3. Subclinical congenital toxoplasmosis at birth

In general, the majority of patients with congenital toxoplasmosis do not present with overt symptoms on routine neonatal examination, and therefore are described as having a subclinical presentation [84,85,86]. If there is a high suspicion of congenital toxoplasmosis, additional investigations can be used to identify more subtle findings that support a diagnosis, such as an ophthalmology review for retinochoroiditis or retinal scars, lumbar puncture for increased levels of protein in the cerebrospinal fluid, and neuroimaging for intracranial calcifications and hydrocephalus [84]. One study showed that only 2 of 52 patients with confirmed congenital toxoplasmosis were identified on routine neonatal examination, and that upon further investigation, 40% of the patients were found to have retinal or brain abnormalities [84].

4. Late manifestations of congenital toxoplasmosis

Regardless of whether patients have apparent or subclinical disease at birth, they remain at continuing risk of developing late manifestations of congenital toxoplasmosis, especially if they do not receive adequate or timely treatment [86]. Retinochoroiditis is the most common late manifestation of congenital toxoplasmosis, with a peak incidence occurring between 4 and 5 years of age, and can even manifest decades after congenital infection [74,76,86]. A prospective study in France followed patients for up to 22 years (median = 10.5 years), and demonstrated that, within this time, 29.8% of patients developed at least one ocular lesion and of these, 33.8% went on to have new or recurrent lesions up to 12 years after the initial lesion was identified [74,90]. The literature suggests that bilateral visual impairment is rare among the European population [74,90]. Comparatively, ocular manifestations are both more severe and more frequent among South American patients, with retinal lesions reported in 60% to 80%, and more than 50% of patients experiencing recurrent active disease [74,91]. In general, congenital toxoplasmosis is thought to be a greater risk factor for vision loss compared to acquired disease, with macula lesions and bilateral involvement being more common in congenitally infected patients [74].

Late neurological manifestations are much less common than retinal lesions and are estimated to affect 10–15% of European and North American patients with congenital toxoplasmosis [91]. Following the trend seen in ocular disease, the prevalence and severity of neurological sequelae seems to be much greater among South American children, as up to 50% of patients with early subclinical disease go on to develop neurological deficits [74,92]. Neurological manifestations can range from mild cognitive impairment to seizures, motor and cerebellar dysfunction, microcephaly, and severe intellectual disability [74,92,93]. Sensorineural hearing loss, bilateral deafness, and endocrinological conditions such as precocious puberty and growth restriction are other late manifestations of congenital toxoplasmosis [58,94]. Although congenital toxoplasmosis can lead to devastating consequences, the results from one study suggest that, when treated, the condition may have little impact on the quality of life of affected individuals [91].

## 4. Psychiatric Diseases and Behaviors Associated with Infection

Chronic latent infection with *T. gondii* was previously thought to be a benign condition, causing harm only in the event of reactivation. However, a large body of the recent literature suggests that chronic infection may be associated with a range of psychiatric and behavioral changes in humans [95]. Evidence of the parasites’ ability to manipulate host behavior is most notable in rodents, who may exhibit the “fatal attraction” phenomenon [96]. This describes a curious change in the behavior of infected rodents who display attraction to the smell of felines, their natural predators [96]. While many studies support the fatal attraction theory [97,98,99,100,101], one recent study refutes the idea and instead suggests that *T. gondii* alters the fear response of mice to all threatening situations, with no specificity for felines above other predators [96]. While the popular theory of fatal attraction is controversial, there is little doubt that *T. gondii* can impact the behavior of rodents [96]. Arguably the more important question is whether the parasite can manipulate the behavior of millions of infected humans. Interestingly, a study involving university students demonstrated a significant, gender-dependent association between *T. gondii* infection and the ranking of the pleasantness of cat urine, with infected men rating cat urine as more pleasant-smelling compared to non-infected men, whereas the opposite was true for women [102].

### 4.1. Personality

Moving beyond the fatal attraction phenomenon, the literature suggests that *T. gondii* infection may alter human personality on a larger scale [103]. Studies have found that infected individuals have significant differences in personality traits compared to uninfected individuals, and that these differences become more pronounced with an increased length of infection [103,104,105]. The results from these studies are heterogenous, but highlight significant and often opposing gender differences in *T. gondii*-associated personality traits: seropositive women tended to score lower on narcissism and were more self-controlled, warm-hearted, outgoing, altruistic, trusting, and accepting compared to seronegative women, while seropositive men were less self-controlled and generous, had fewer and less intense relationships and were more suspicious and jealous compared to seronegative men [103,104,106,107,108].

### 4.2. Risky Behavior

Several studies have investigated how personality traits associated with *T. gondii* infection may apply to human behavior on a more practical level. In particular, the literature shows that *T. gondii* infection is significantly associated with risky behaviors including substance abuse and overdose, excessive alcohol consumption, suicide attempts, and disregard for reasonable safety precautions (e.g., not wearing a helmet) [109,110,111,112]. Furthermore, two separate meta-analyses have linked *T. gondii* seropositivity with a significantly increased risk of having a traffic accident [111,113]. However, not all risky behavior results in negative outcomes, with one study demonstrating that *T. gondii*-infected college students were more likely to major in business, and *T. gondii*-infected attendees to entrepreneurial events were more likely to have started their own business [110]. Furthermore, at a national level, *T. gondii* seroprevalence was a significant positive predictor for entrepreneurial activity, with fewer participants reporting a “fear of failure” preventing new business ventures in world regions with high seroprevalence [110].

### 4.3. Schizophrenia

Schizophrenia is a serious psychiatric disease that usually presents in early adulthood and affects approximately 1% of the global population [114]. It is characterized by a combination of positive symptoms such as hallucinations and delusions, along with negative symptoms such as avolition, alogia, and anhedonia, which collectively may result in significant disability [114]. Current research indicates that schizophrenia has a multifactorial etiology, with several identified genetic and environmental risk factors [115]. Among these environmental risk factors, infections, and, in particular, *T. gondii* infection, have been suggested as playing a role in the development of schizophrenia [95,115].

Numerous studies have found a statistically significant association between *T. gondii* infection and schizophrenia, with two meta-analyses finding the odds ratio (OR) to be up to 2.7 [116,117]. While an OR of 2.7 represents a moderate association, this is greater than those found for almost all other genetic and environmental associations with schizophrenia [95,118]. One study has demonstrated that maternal exposure to *T. gondii* that results in high (≥1:128) maternal *Toxoplasma* IgG titers is associated with increased risk of schizophrenia or schizophrenia spectrum disorders in adult children (OR = 2.61, 95% confidence interval = 1.00–6.82) [119], while a study of neonates has showed that high *Toxoplasma* IgG levels are significantly associated with the development of early-onset schizophrenia (OR = 1.79) [120].

#### 4.3.1. Etiological Links

It has been proposed that changes in cell signaling pathways in the brain as a result of *T. gondii* infection could play a role in the pathogenesis of schizophrenia. The “revised dopamine hypothesis” purports that hyperactive dopamine transmission in the mesolimbic region of the brain contributes to positive symptoms of schizophrenia, while hypoactive dopamine transmission in the prefrontal cortex contributes to negative symptoms [121]. This is of particular interest as *T. gondii* has genes that promote the expression of the enzyme tyrosine hydroxylase, which produces levodopa, a precursor for dopamine [122]. Indeed, separate studies showed that dopamine levels were 14% greater in the brains of chronically infected mice compared to controls [123], and *T. gondii*-infected mammalian cells produced three times more dopamine than uninfected cells [124]. Dopamine is perhaps the most researched neurotransmitter affected by *T. gondii* [125], but others, such as serotonin [125], gamma-aminobutyric acid [126], glutamate [127], noradrenaline [125], nitric oxide [128], and kynurenic acid [129,130], have also been reported [131].

#### 4.3.2. Clinical Manifestations

Several studies in the literature support that concurrent infection with *T. gondii* is associated with an increase in the severity of symptoms in schizophrenia [119,120,132,133]. In particular, seropositive patients have more severe positive and negative symptoms [134,135,136,137], remain in hospital for longer [136], are more likely to have a continuous disease course [138], and have an increased risk of mortality compared to seronegative schizophrenic patients [139]. A Voxel-based morphometry imaging study supported these clinical observations by demonstrating that gray matter volume is significantly reduced in seropositive patients compared to controls [140]. 

#### 4.3.3. Effect of *T. gondii* Treatment upon Schizophrenia

If *T. gondii* has a role in the pathogenesis of schizophrenia, as some literature postulates, is it possible to treat the symptoms of schizophrenia by targeting the concurrent *T. gondii* infection? Four separate randomized controlled trials (RCTs) have attempted to answer this question, and none so far have demonstrated that adjuvant anti-parasitic therapy with azithromycin [141], trimethroprim [142], artemisinin [143], or artemether [144] produces any significant improvement in schizophrenia symptoms [141,145]. However, none of the RCTs trialed drug regimens that have reported effectiveness against *T. gondii* in experimental models [145].

#### 4.3.4. Effect of Psychotropic Treatment upon *T. gondii*

While anti-parasitic drugs have not be shown to improve symptoms in seropositive patients, some anti-psychotic and mood-stabilizing drugs have activity against *T. gondii* [146]. In particular, haloperidol and valproate were found to have a strong inhibitory effects on *T. gondii* growth in vitro [147]. These findings were supported by an in vivo study of infected rats, which demonstrated that haloperidol or valproate were as effective as standard anti-parasitic treatments in preventing *T. gondii*-induced behavioral and cognitive changes [99]. However, another in vitro study found that while several anti-psychotics, particularly fluphenazine and zuclopenthixol, had high anti-parasitic activity, valproate had no effect on *T. gondii* growth [148]. The authors of this study proposed that valproate was only effective in preventing cellular invasion, suggesting that the developmental stage of the parasite and the timing of infection impacted the effectiveness of the psychotropic medications [95,148]. Both haloperidol and valproate inhibit calcium transport, which is essential for tachyzoite’s invasion of host cells, providing a possible explanation for why these drugs might be effective at this stage of the *T. gondii* lifecycle [147].

### 4.4. Bipolar Affective Disorder

Bipolar affective disorder (BPAD) is characterized by recurring episodes of mania or hypomania interspersed with periods of depression or normal mood [149]. The global lifetime prevalence of BPAD is estimated at 2.4%, with symptoms typically beginning in the late teens [149,150]. The disorder is associated with a significant burden of illness, and is especially devastating due to the increased risk of suicide, which has been estimated as being up to 30% greater than in the general population [149]. While the etiology of BPAD is known to have a large genetic component, environmental risk factors, including infections, have also been demonstrated to contribute to its development [151].

Compared to schizophrenia, there have been fewer studies investigating the potential role of *T. gondii* infection in the etiology of BPAD. Several case–control studies have demonstrated a significant association between *T. gondii* seropositivity and BPAD, with an odds ratio ranging from 2.17 to 3.52 according to different populations, including samples from Ethiopia (OR = 3.0), France (OR = 2.17), the United States (OR = 2.4), and Turkey (OR =3.52) [152,153,154,155]. While several meta-analyses also supported a significant association between BPAD and *T. gondii* infection (OR = 1.26–1.52), the results were heterogenous and the authors all suggested the need for further research [112,156,157]. Indeed, one case–control study found no statistically significant association between BPAD and *T. gondii* seropositivity, while another study found no association between maternal *Toxoplasma* IgG titer during the third trimester and BPAD, which had previously been found to confer an increased risk of schizophrenia [158,159]. Although BPAD has been comparatively understudied, to date, the general trend in the literature supports an association between *T. gondii* infection and the disorder [112,152,153,154,155,156,157].

#### Clinical Manifestations

Few studies have provided insight into how *T. gondii* infection alters the clinical manifestations of BPAD, with one study demonstrating that seropositivity is associated with later onset and with more frequent lifetime suicide attempts [160]. Of all psychiatric conditions, BPAD and schizophrenia have two of the highest rates of suicide [161]. It is particularly concerning, therefore, that some studies have found that concurrent *T. gondii* infection in patients with schizophrenia or BPAD is associated with increased suicidality [160,162]. Studies have also shown that *T. gondii* infection alone is significantly associated with an increased risk of suicide attempts [155]. Furthermore, as with schizophrenia, many of the psychotropic medications used to treat BPAD have anti-parasitic activity [148,156]. Using this discovery, one study was able to show that *T. gondii*-seropositive patients with BPAD experienced an increased frequency of depressive episodes over their lifetime when treated with a psychotropic drug with no activity against *T. gondii* compared to patients treated with drugs with known activity [160].

### 4.5. Other Psychiatric Conditions

Several other psychiatric and neurodegenerative disorders, including obsessive compulsive disorder (OCD), Alzheimer’s disease, and depression, have been investigated for an association with *T. gondii* infection, as was recently reviewed [163]. Several studies have found that OCD is significantly associated with *T. gondii* seropositivity [164,165], with an OR of 1.96 found by one systematic review and meta-analysis [166]. There is no clear association between seropositivity and Alzheimer’s disease, with some studies and reviews suggesting that *T. gondii* infection is a risk factor for this disease [167,168,169], while many other studies have found no statistically significant association [170,171,172]. The relationship between *T. gondii* and depression is also controversial in the literature, although most sources agree that depression is not associated with *T. gondii* [173,174,175,176].

## 5. Treatment

Current anti-parasitic agents mostly target the metabolically active tachyzoites, not the dormant bradyzoites [177,178]. The current gold standard for *T. gondii* treatment is a combination of pyrimethamine and sulfadiazine, although the failure rates for all known treatment regimens remain quite high, and none are able to eradicate latent disease [177,178]. Furthermore, pyrimethamine and sulfadiazine treatment is associated with potentially severe side effects, mostly due to the allergic reactions to sulfadiazine and hematologic toxicity associated with pyrimethamine [177]. In order to mitigate these effects, folinic acid supplementation is recommended for all treatment regimens, including pyrimethamine [177]. Table 1 provides detailed descriptions of the treatment approaches in different clinical settings.

### 5.1. Acute Toxoplasmosis in Immunocompetent Patients

Acute infection with *T. gondii* is usually subclinical and self-limited, generally requiring no treatment unless symptoms are unusually severe or prolonged [177,178]. Pyrimethamine and sulfadiazine is the standard approach to treatment despite the limited evidence to support its use in immunocompetent patients [177]. Furthermore, one RCT demonstrated that trimethoprim and sulfamethoxazole treatment for 1 month was effective in resolving lymphadenitis in patients with an acute infection [179].

### 5.2. Acute Toxoplasmosis in Immunocompromised Patients

Extracerebral manifestations of toxoplasmosis in immunocompromised patients are less common and are treated with the same regimens as for cerebral toxoplasmosis, although the clinical response rate may be lower [177,180]. Without intervention, cerebral toxoplasmosis may be fatal, and even delays in the onset of treatment can lead poor clinical outcomes [177]. For this reason, immunocompromised patients with signs of cerebral toxoplasmosis should be treated empirically, with close observation for treatment failure [177]. Several systematic reviews and meta-analyses have shown no significant difference in efficacy between pyrimethamine and sulfadiazine, trimethoprim–sulfamethoxazole, and pyrimethamine–clindamycin in treating cerebral toxoplasmosis among the HIV-infected population [177,178,181,182,183].

**Table 1:** Treatment regimens for diseases caused by *T. gondii* infection [88,89,177,178,181,182,183,184,185,186,187].

Type of Disease	Antimicrobial Drug Regimen	Dose		Duration of Treatment
Acute toxoplasmosis in immunocompetent patients	Pyrimethamine–sulfadiazine	*Pyrimethamine* Loading dose for first 1–2 days: 100 mg daily Followed by 25–50 mg daily + *Sulfadiazine* 1 g every 6 h + *Folinic acid* 10–20 mg daily		4–6 weeks
Toxoplasmosis in immunocompromised patients Includes cerebral toxoplasmosis and extracerebral manifestations, Excludes ocular toxoplasmosis	Pyrimethamine–sulfadiazine	**Induction therapy** *Pyrimethamine* Loading dose: 200 mg Followed by 75 mg daily (if weight < 60 kg, then 50 mg daily) *+ Sulfadiazine* 1.5 g every 6 h (if weight < 60 kg, then 1 g every 6 h) *+ Folinic acid* 10–50 mg daily	**Maintenance therapy** *Pyrimethamine* 50 mg daily (if weight < 60 kg, then 25 mg daily) *+ Sulfadiazine* 1 g every 6 h (if weight < 60 kg, then 0.5 g every 6 h) *+ Folinic acid* 10–25 mg daily	Induction therapy is given for 6 weeks to manage acute disease. Maintenance therapy is continued for as long as patient remains immunosuppressed.
	Trimethoprim–sulfamethoxazole	*Trimethoprim–sulfamethoxazole combined capsule* 10 mg/50 mg/kg/day in divided doses	* Trimethoprim-sulfamethoxazole combined capsule * 5 mg/25 mg/kg/day in divided doses	
	Pyrimethamine–clindamycin	* Pyrimethamine * Loading dose: 200 mg Followed by 75 mg daily (if weight < 60 kg, then 50 mg daily) * + Clindamycin * 600 mg every 6 h * + Folinic acid * 10–50 mg daily	*Pyrimethamine* 50 mg daily (if weight < 60 kg, then 25 mg daily) *+ Clindamycin* 600 mg every 8 h *+ Folinic acid* 10–25 mg daily	
Ocular toxoplasmosis In non-pregnant and immunocompetent patients	Pyrimethamine–sulfadiazine	* Pyrimethamine * Loading dose: 100 mg Followed by 25–50 mg daily * + Sulfadiazine * Loading dose: 2 g Followed by 500 mg–1 g every 6 h * + Folinic acid * 10–20 mg daily		Treatment is given until resolution of active retinal lesion (usually 4–6 weeks).
	Trimethoprim–sulfamethoxazole	*Trimethoprim*–*sulfamethoxazole* 160 mg/800 mg combined capsule twice daily		
Toxoplasmosis acquired in pregnancy Maternal infection < 14 weeks gestation, with no confirmed fetal infection	Spiramycin	1 g, every 8 h		Treatment should be continued until delivery.
Toxoplasmosis acquired in pregnancy Maternal infection at >14 weeks gestation OR confirmed fetal infection	Pyrimethamine–sulfadiazine	* Pyrimethamine * First 2 days: 100 mg/day Remaining time: 50 mg/day * + Sulfadiazine * Maternal weight < 80 kg: 1 g every 8 h Maternal weight > 80 kg: 1 g every 6h * + Folinic acid * 10–20 mg/day		
	Pyrimethamine–sulfadoxine	*Pyrimethamine*–*sulfadoxine combined capsule* 25 mg/500 mg combined capsule, 2 capsules per week *+ Folinic acid* 25 mg twice per week		
Postnatal congenital toxoplasmosis	Pyrimethamine–sulfadiazine	* Pyrimethamine * First 2 days: 1 mg/kg twice daily Next 2–6 months: 1 mg/kg daily (consider 6 months for symptomatic congenital toxoplasmosis) Remaining time: 1 mg/kg/day 3 times a week * + Sulfadiazine * 50 mg/kg twice daily * + Folinic acid * 10 mg 3 times per week		Treatment should be initiated promptly and continued for a minimum of 12 months. Long-term follow-up is also necessary to monitor for late manifestations.
	Pyrimethamine–sulfadoxine	* Pyrimethamine * 0.875 mg/kg once per week * + * *Sulfadoxine* 17.4 mg/kg once per week * + Folinic acid * 25 mg twice per week		

### 5.3. Ocular Toxoplasmosis in Non-Pregnant and Immunocompetent Patients

The combinations of pyrimethamine and sulfadiazine, and trimethoprim and sulfamethoxazole are both used to treat ocular toxoplasmosis [48,177,184,186,188]. However, a Cochrane review in 2016 found no clear evidence that antimicrobial treatment improved visual outcomes [189]. Topical, systemic, and/or intravitreal corticosteroid drugs may be used in conjunction with an antimicrobial regimen [64], and although they can be helpful for cases with severe inflammation, their use is less well supported in the literature [177,190]. Furthermore, these agents should not be used without antimicrobial therapy, as corticosteroid monotherapy can promote parasite growth and result in severe disease that leads to serious vision impairment and even blindness [177]. Despite being the most common manifestation of *T. gondii* infection, there is a clear lack of evidence guiding the effective treatment of ocular toxoplasmosis, as highlighted by the American Academy of Ophthalmology, which concluded in 2012 that “There is a lack of level I evidence to support the efficacy of routine antibiotic or corticosteroid treatment for acute [toxoplasmic retinochoroiditis] in immunocompetent patients” [191].

### 5.4. Toxoplasmosis During Pregnancy

Antimicrobial treatment is not necessary for immunocompetent pregnant mothers with latent toxoplasmosis, and is only recommended for maternal toxoplasmosis acquired during pregnancy [177]. The antimicrobial regimen differs according to the gestational age of the fetus, due to both the teratogenic properties of pyrimethamine and sulfadiazine in early pregnancy, and the effect of gestational age upon the risk of transmission and the severity of resultant congenital toxoplasmosis [73,74,88,177,187,192]. Spiramycin is used for maternal infection at less than 14 weeks gestation, while pyrimethamine and sulfadiazine therapy is used for maternal infection of greater than 14 weeks gestation [88,177,187]. However, as spiramycin does not effectively treat established fetal infection, confirmation of fetal infection via amniotic fluid PCR indicates that the antimicrobial approach should be switched to pyrimethamine and sulfadiazine [88,177,187]. Conversely, if the fetal ultrasound is normal and amniotic fluid PCR is negative for a maternal infection of greater than 14 weeks gestation, the pyrimethamine and sulfadiazine regimen can be switched to spiramycin, which is better tolerated and has a documented safety profile in pregnancy [88,177,187]. Other common anti-parasitic regimens, such as trimethoprim and sulfamethoxazole, or pyrimethamine and clindamycin have not been validated for safety or effectiveness in pregnancy [177].

### 5.5. Congenital Toxoplasmosis

Due to the potentially devastating consequences of congenital toxoplasmosis, treatment should be initiated as soon as possible in diagnosed or high-risk newborns, irrespective of maternal treatment during pregnancy or the absence of symptoms [89,187,193]. Pyrimethamine and sulfadiazine is the first-line treatment, although the combination of pyrimethamine and sulfadoxine appears to be well tolerated in newborns and is used as an alternative [177,194,195].

## 6. Prevention

The high global burden of disease associated with *T. gondii* infection combined with the lack of measures to eradicate latent disease emphasizes the need for effective prevention measures [196]. Individuals can minimize their risk of exposure through safe food handling practices, including (1) avoiding the consumption of raw meat; (2) killing *T. gondii* in meat by either cooking to an internal temperature of 66 °C or freezing at −12 °C before cooking; (3) washing or peeling fruit and vegetables prior to ingestion; and (4) ensuring utensils and surfaces that have come into contact with raw meat or unwashed fruits and vegetables are cleaned thoroughly [177,196,197,198,199]. High-risk individuals, including pregnant women and immunocompromised individuals, can safely own a cat, but should avoid contact with cat litter [18,25,177,196,197]. Gloves should be worn while changing cat litter and while gardening, followed by handwashing [177,196,197].

Further measures may be necessary to protect high-risk individuals. One study found that only 28% of *T. gondii* infections acquired during pregnancy could be avoided by following preventative strategies [200]. One solution is to implement prenatal serological screening of all pregnant women, as occurs in France and Austria [19,177,196]. Monthly prenatal screening was demonstrated to significantly decrease fetal infection and improve clinical outcomes for infected children in one study conducted in France, although the cost-effectiveness of such programs is controversial and has prevented other countries from implementing systematic screening [196,201]. Yet, prenatal screening has been found to be more cost-effective compared to neonatal screening, and several studies suggest that prenatal screening is cost-effective and should be implemented more widely [202,203,204]. One study showed that the prenatal screening program in Austria was cost-saving, while another study used a mathematical model to demonstrate that implementing the French model of prenatal screening would be cost-saving in the United States [203,204].

Preventative measures are also considered for immunocompromised patients, especially those persons infected with HIV, in whom latent *T. gondii* infections can reactivate [177]. In order to prevent reactivation, prophylaxis using the same anti-parasitic drugs as for immunocompromised patients with active disease is recommended for *T. gondii* seropositive HIV-infected individuals when their CD4-positive T cell count is less than 100/mm^3^ and should be continued until the count has remained above 200/mm^3^ for a minimum of 6 weeks on antiretroviral therapy [177].

Finally, there are public health measures that can be used to protect communities on a larger scale. To prevent waterborne infection, standard municipal water treatments involving coagulation, flocculation, and settling prior to filtration are effective at removing *Giardia* and *Crytosporidium* cysts, and thus should be effective at removing comparatively larger *T. gondii* oocysts [205,206]. Implementing farming practices can prevent infection in food animals, including housing animals indoors, the provision of clean water and sterilized feed, and strictly limiting animal and feed contact with cats, rodents, and birds. While controlled indoor farming has been shown to be effective, it is much more challenging to prevent infection in animals reared outdoors [196]. A vaccine against *T. gondii* for cats or livestock would be of great benefit in combating toxoplasmosis. Currently, only one vaccine is licensed in Europe and New Zealand for use in sheep to help prevent abortions from congenital toxoplasmosis, with further advancements proving challenging due to the complex lifecycle, multiple strains, and genetic diversity of *T. gondii* [207]. 

## 7. Conclusions

*T. gondii* infections have the potential to cause serious disease in humans, and recent research suggests there may also be behavioral and psychiatric implications. None of the available treatment options are curative, highlighting the importance of implementing effective preventive strategies, and emphasizing the value of research into vaccines and curative anti-parasitic drugs.

## Data Availability

Data sharing is not applicable to this article. No new data were created or analyzed in this work.
